# Comparative study of NiO/CuO/Ag doped graphene based materials for reduction of nitroaromatic compounds and degradation of dye with statistical study

**DOI:** 10.1038/s41598-024-51342-x

**Published:** 2024-01-24

**Authors:** Gayatri Pandey, Nidhi Singh, Nitesh Rajput, Mahesh Kumar Saini, S. L. Kothari, Jagdish Prasad, Narendra Pal Lamba, Manmohan Singh Chauhan

**Affiliations:** 1 Amity University Rajasthan, Jaipur, India; 2 Department of Information System, North Eastern University, Boston, USA

**Keywords:** Environmental sciences, Chemistry

## Abstract

In the present work, the Nickel oxide (rGO–NiO), Silver (rGO–Ag), Copper oxide (rGO–CuO) doped Graphene Oxide are reported for catalytic reactions. A comparative study for catalytic activities of these materials are performed with nitroaromatic compound 4-nitroaniline and the results are statistically studied by using univariate analysis of variance and Post Hoc Test through Statistical Package for Social Sciences and it is observed that CuO doped Graphene material is showing better catalytic activity in minimum time. So, further research has been focused on the catalytic acitivity of rGO–CuO only and it is found that it is efficient in reducing other nitro compounds also such as Picric acid and Nitrobenzene. Dye degradation of Methylene blue is also performed using CuO decorated Graphene material and significant changes were observed using UV spectroscopy. The characterization of rGO–CuO is done with Fourier-transform Infrared Spectroscopy, Powder X-ray Diffraction, Thermogravimetric Analysis, Scanning Electron Microscope and Transmission Electron Microscopy.

## Introduction

Currently, Researchers and environmentalists are concerned about nitroaromatic compounds and dyes. In order to decompose these contaminants without further harming the environment, researchers are looking for improved catalytic system. The nitroaromatic compounds such as 4-Nitroaniline (4-NA), Picric Acid (PA), Nitrobenzene have been used in numerous chemical reactions and they have wide applications in drug, pesticides, insecticides, and corrosion industries. As an industrial effluent, they can pollute water bodies and have carcinogenic effects on human beings also. The nitroaromatic compounds have electron-withdrawing character which give them strong xenobiotic properties and in turn make them difficult to biodegrade. So, di and tri nitro compounds are difficult to biodegrade. Due to their distinctive electron-withdrawing character, nitro compounds have a highly deficient π-electron system, by generating a strong xenobiotic character. Nitroaromatic and other nitrogen containing organic compounds are resistant to biodegradation. Due to poor electron density on nitro group the electrophilic attack on aromatic ring becomes difficult which is the first step in the degradation^1^. In the textile and drug industries dyes such as Methylene Blue (MB) are used from decades and with numerous applications are considerable but its hazards are also seeking interest of researchers. As research specify the textile industries releases a large amount of untreated dye in the water bodies which can cause serious health effects on aquatic life, human beings and animals. It has toxic properties, that can cause cancer, respiratory distress, can cause serious environmental issues and also it is non-biodegradable^2^.

In this context, researchers main concern was bounding to the development of catalysts with high catalytic activity, a very promising 2D material that is Graphene is used to synthesize Graphene Oxide which is seeking attention of many researchers since the properties of it is very promising such as having high surface area and many more^3^. Agglomeration is a key disadvantage of employing bare composites. As a result, some sort of stabilizer is required. So, the Graphene Oxide with very promising properties has piqued scientists' interest. Graphene oxide and many other composites such as those made up of Titanium and Zinc possesses intriguing applications in thermal, mechanical and in addition to electrical properties also^4^, as well as a vast surface area with a variety of functional groups, allowing for chemical changes and making it a promising research option^5,6^. These composites have various applications like in catalysis^5^, sensors^7,8^, and photocatalysis^9^, for the reduction process in organic synthesis, toxic metal ion removal^10,11^, cellular imaging^12,13^, drug delivery^12^, and many other features have been documented in the literature^14–18^. Graphene based materials can also be used as efficient heterogeneous catalysts^19,20^ and its extraordinary conductivity can aid electron transport during transformations^21^. As a result, researchers found that Graphene based materials with metal doped on Graphene oxide can be proved as perfect catalysts in many reactions, thereby increasing reduction efficiency and in turn can be efficiently used in waste water treatment^22^. Indeed, the interaction of less reactive composites with graphene results in highly active hybrid composite catalysts^23^. Nowadays, for dealing with global energy crisis graphene based materials are also used in photocatalytic field also^24,25^. So, due lesser coordination number and more reach to some efficient surface atoms, these composites are good catalysts for the degradation of pollutants when compared to bulk material^26^. Because aggregation of particles reduces catalytic activity and reusability, approaches to promote particle stability and avoid aggregation are needed^27,28^. The majority of these composites are immobilised on the surface of certain supporting materials. These substances are commonly utilised in the decomposition of organic contaminants. Carcinogenic amines are formed when nitro Comprehensive Organic Synthesis are reduced in aquatic bodies’ sediments^29^. Many colours including dyes have been shown to be made from cancer causing organic compounds which may result in severe diseases such as cancer, Hepatocarcinoma and many more diseases related to animals and humans^30^. It is requires to time to introduce novel method and ways which reduce and degrade compounds without causing any harm to human being and environment^31–34^.

This study focuses on synthesis and applications of metal (CuO, Ag, NiO) ion supported reduced Graphene oxide materials for the curtailment of aromatic nitro compounds such as 4-Nitroaniline, Picric Acid, Nitrobenzene and in dye degradation of Methylene Blue using standard techniques. Also, a comparative study between Graphene oxide composites of Nickel, Silver and Copper has been shown in this paper and best results for determining the minimum reaction time in the reduction of 4-Nitroaniline and then other nitro compounds and Methylene Blue dye are also reduced using the better catalyst (rGO–CuO) after the full factorial analysis on the data of 4-Nitroaniline using Statistical Package for Social Sciences(SPSS).

## Materials and methods

The components used are purchased from Sigma-Aldrich and are as follows: Graphite powder, Potassium Permanganate, Sulphuric acid, Phosphoric acid, Hydrogen Peroxide, Nickel Chloride and Sodium Borohydride.

**Figure 1:**
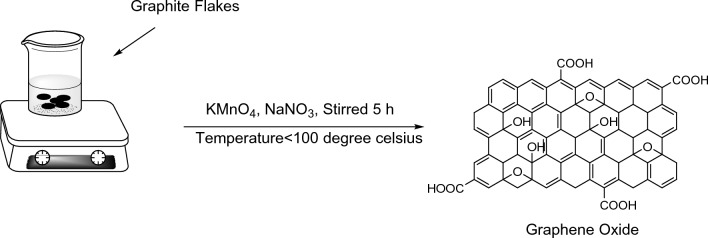
Preparation of graphene oxide from graphite flakes.

*Graphene oxide (GO) preparation:* We have used modified Hummer’s Method (Fig. 1) for the synthesis of GO^35,36^. In an ice-bath, a beaker was placed and Conc. H_2_SO_4_ (100 ml) was added into it and then 5 g Graphite powder is mixed into the beaker. After that for 2–3 min the mixture was stirred then, Potassium Permanganate (120 g) was delivered into the mixture and stirred for another 3, after that Sodium Nitrate (2.5 g) is added in it. Until the prepared mixture became greenish-black and slightly thick it was stirred foe almost 2.5 h. After that the prepared reaction mixture was placed in water bath at 35 °C and then distilled water was added slowly into the reaction mixture. The final solution obtained was stirred for 3 h and the temperature was kept 80 °C. The progress of reaction is observed with the appearance of reaction mixture changed to slightly yellowish. H_2_O_2_ (200 ml) was used for the treatment of prepared final mixture and resultant mixture was filtered using whatman filter paper. Then after filtration obtained mixture was mixed with distilled water and sonicated for 1 h. After that the Graphene oxide solution was centrifuged at speeds ranging from 2000 to 5000 rpm. At last, the obtained precipitate is dried and ground in fine powder form to get Graphene oxide (Fig. 1).

**Figure 2:**
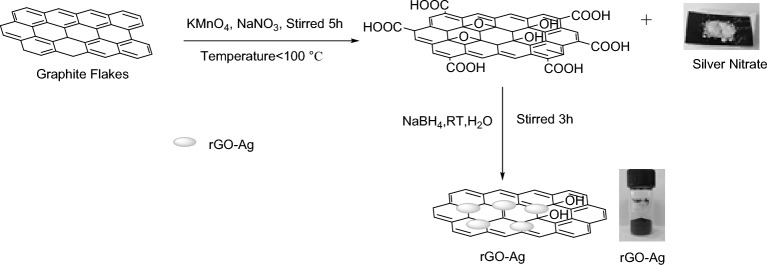
Preparation of rGO–Ag.

*Preparation of Silver decorated reduced Graphene Oxide(rGO–Ag):* GO (1 g) was introduced to 400 mL of distilled water in a beaker. Graphene oxide powder and distilled water was sonicated for 10 min to obtain the homogenous mixture. After that, 1 g Silver Nitrate was added in it and allowed to stir for 20 min. A solution of distilled water (50 ml) and Sodium borohydride NaBH_4_ (1 g) was prepared in a beaker. Using a glass rod, the solution was swirled until the NaBH_4_ was completely dissolved in distilled water and then it was added drop-wise in mixture of GO and Silver Nitrate solution with continuous stirring and after full addition of NaBH_4_ solution the mixture was allowed to stir for 2 h at room temperature. The reaction mixture was filtered and washed many times with distilled water. Finally, to obtain Ag decorated reduced Graphene oxide (Fig. 2) the resulting precipitate was dried in an oven for 8–10 h^37,38^.

**Figure 3:**
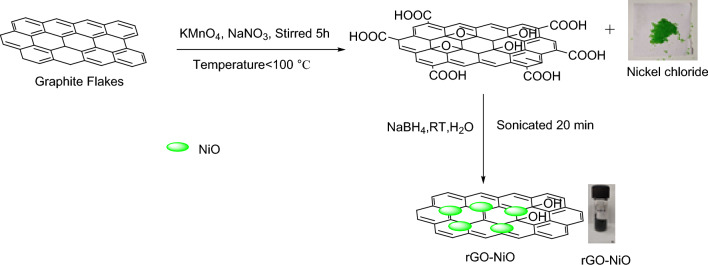
Preparation of rGO–NiO.

*Preparation of nickel oxide decorated reduced graphene Oxide(rGO–NiO):* The distilled water (250 ml) and Graphene oxide (500 mg) were taken in a conical flask(500 ml). Then, the obtained mixture was sonicated for 20 min. After sonication, NiCl_2_.6H_2_O (500 mg) delivered into the solution of Graphene oxide and for 30 min it was again sonicated. After that, solution of NaBH_4_ (185 mg dissolved in 100 ml water) was added in it. A dark precipitate was found to develop in a couple of minutes after addition of sodium borohydride solution. Finally, Nickel decorated reduced Graphene oxide (Fig. 3) was obtained as precipitate. The obtained precipitate was filtered, and using distilled water it was 4–5 times to remove the impurities. At last, it was placed in oven for 6 to 8 h for drying the composite^38,39^.

*Preparation of copper oxide (CuO):* In a beaker, solution of NaOH (5 gm NaOH is added in 20 ml water) was added into the solution of CuSO_4_ (2gm CuSO_4_ in 20 ml water) dropwise until the precipitate of bluish colour was formed and separated completely. Then, it was stirred on magnetic stirrer until dark black precipitate was formed. After that, it was filtered and dried in oven.

**Figure 4:**
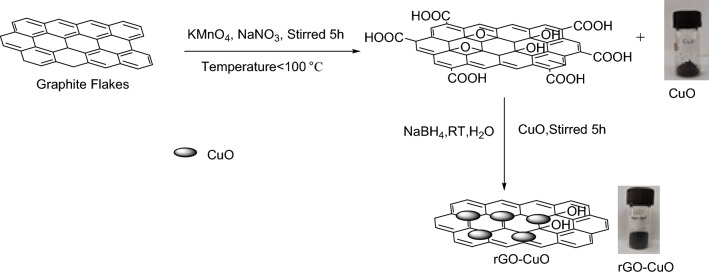
Preparation of rGO–CuO.

*Preparation of copper oxide decorated graphene oxide (*rGO–CuO*):* Graphene oxide (1 gm) is added in a beaker containing 200 ml distilled water. Then, the resultant mixture is sonicated for 10 min. In an another beaker, CuO(400 mg) was added in distilled water(100 ml) and sonicated for 10 min. Then, sonicated Graphene oxide mixture was added in sonicated Copper oxide solution, after that NaBH_4_ (50 mg) was added into it. The resultant reaction mixture was allowed to stirred for 4 h. After that, it was filtered and ethanol was used for washing it several times and then it was placed in oven for 8–10 h for drying at 60–80 ° temperature to get Copper decorated reduced Graphene oxide (Fig. 4)^39^.

*Catalytic performance of prepared materials in reduction of Nitro-aromatic Compounds and Dye:* Prepared material is added in reaction mixture of water and relevant nitroaromatic compounds (0.1 mmol) or dye (100 ppm) and allowed to stir at room temperature. The progress of reaction is checked by UV visible spectroscopy by taking time basis plot and thin layer chromatography.

*Characterization:* The structure and surface properties were determined by XRD, TEM, SEM–EDX, FTIR. The XRD used to determine the powder properties of the synthesized graphene based material, which is observed with Cu Kα radiation and with wavelength 0.15418 nm.

FT-IR study was also done of prepared materials to analyse and observe the peaks of different functional groups with Perkin Elmer instrument. The STA 6000 from Perkin Elmer was used to determine the thermo-gravimetric measurements. The measurements were taken in a Nitrogen atmosphere with the heating rate of 10 °C. The morphology of the synthesized material was measured by the field emission scanning electron microscopy and the measurements were taken using Nova Nano FE-SEM 450 which has the resolution up to 1.6 nm at 1kv. UV–visible spectrophotometer LAMBDA 750 (Perkin Elmer) instrument was used for the residual concentrations of catalytic reduction studies of Nitroaromatics and dye. The uptake of metal ions by catalyst were measured and also to determine the residual concentrations of catalytic reduction and studies of Nitroaromatics and dye the flame atomic absorption spectrophotometer LAMBDA 750 (Perkin Elmer) UV–Vis NIR Spectrophotometer instrument was used.

## Results and discussion

**Figure 5:**
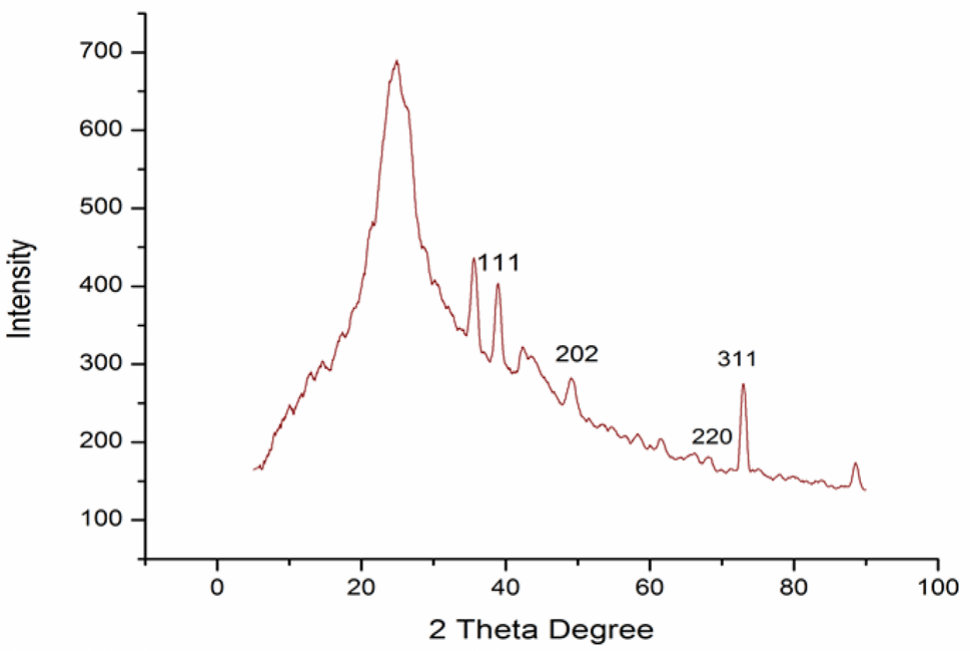
XRD pattern for CuO–rGO powder.

*XRD-* Fig. 5 shows the PXRD patterns of CuO–rGO in which XRD exhibits a broad peak at 2θ = 25°, furthermore, diffraction peaks at 2θ = 28.72^○^(002), 38.99^○^ (111), 42.41^○^ (111), 46.47^○^ (200), 50.53^○^ (202), 53.46^○^ (020), 60.09^○^ (202), 64.06^○^ (220), 66.82^○^ (022), 69.82^○^ (113) and 72.90^○^ (311) which were consistent with the standard XRD data for CuO monoclinic phase of end-centered crystal lattice(JCPDS card no. 89–2530)^40^. The crystalline size is calculated using Scherrer equation (Eq. 1), percent cystallinity is calculated using equation-2 and porosity is calculated using equation-3.The crystalline size is found to be approx. 20 nm, percentage cyrstallinity is found to be 55% and porosity is found to be 99% respectively.

1 $${\text{D}} = { }\frac{{{\text{K}}\lambda }}{{\beta {\text{cos}}\theta }}$$

2 $${\text{\% Cyrstallinity}} = { }\frac{{\text{Area of cyrstalline peaks}}}{{\text{Area of all peaks}}} \times 100{ }$$

3 $${\text{Porosity}} = \left[ {1 - \frac{{\text{Bulk density}}}{{\text{X ray density}}}} \right] \times 100$$

**Figure 6:**
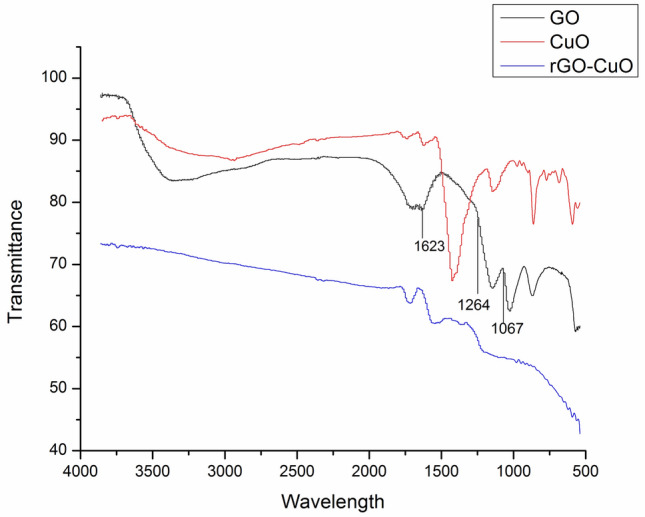
IR spectra of CuO, GO and rGO–CuO.

*FTIR spectroscopy:* FTIR spectra (Fig. 6) of CuO–rGO was observed to determine various functional groups in materials including Graphene oxide, Copper oxide and rGO–CuO. In the FTIR spectrum of GO the peaks obtained at 1264 cm^−1^, 1067 cm^−1^ and 1721 cm^−1^ are attributed to the C–O epoxy stretching, C–O alkoxy stretching and C=O carbonyl stretching vibrations, respectively. The absorption band at 1623 cm^−1^ and 3421 cm^−1^. correspond to the aromatic C=C stretching vibration and O–H stretching vibration. These peaks disappeared in the CuO–rGO nanocomposite catalyst and the intensity of peaks C=O, C–OH and C=O decreases verifying the reduction of Graphene Oxide during the preparation. This implies the reduction of Graphene Oxide along with formation of Nano-composite^5^.

**Figure 7:**
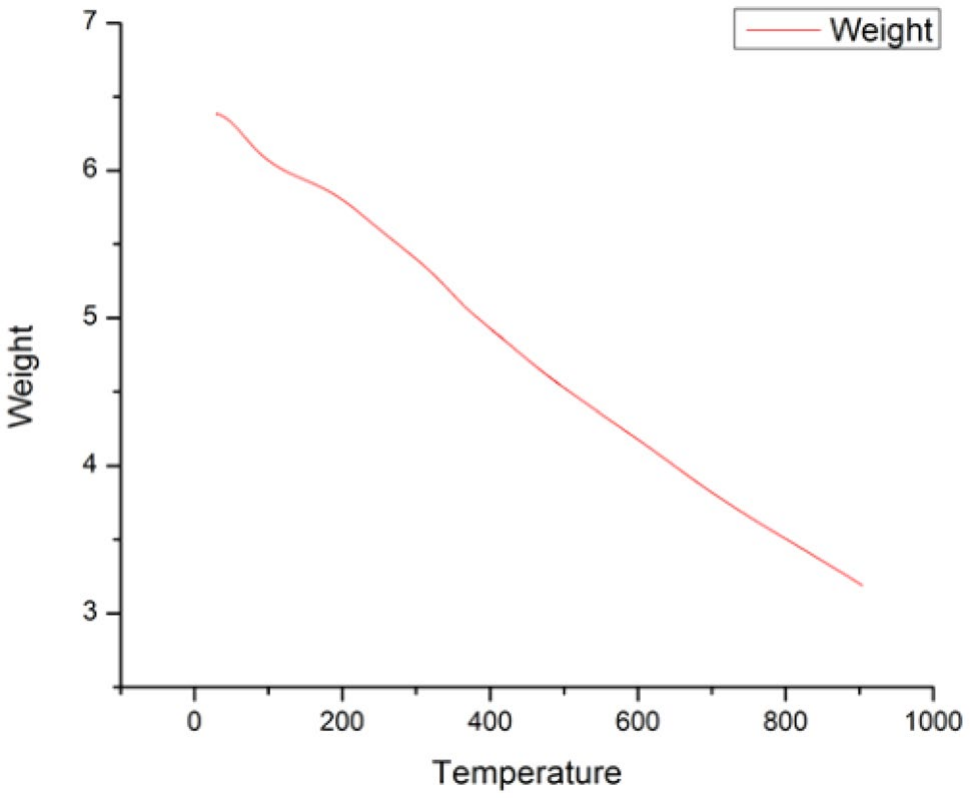
TGA of CuO–rGO.

*Thermogravimetric analysis (TGA):* The catalyst is further characterized by Thermogravimetric Analysis(TGA) was used to test the thermal stability of material under a nitrogen environment with only a temperature ramping of 10 °C/min up to 900 ^○^C. As shown in Fig. 7, 12 wt% loss observed till 150 °C, which is caused by removal of moisture content and the evaporation of intercalated water molecules. Around 38 wt% loss was observed after 150 °C, which may be related to the breakdown of the several functional groups that CuO–rGO contains. We can conclude that the proper formation of nano-composite between Copper Oxide and reduced Graphene Oxide leads to positive synergism with a high temperature stability.

### Scanning electron microscopy (SEM):

**Figure 8:**
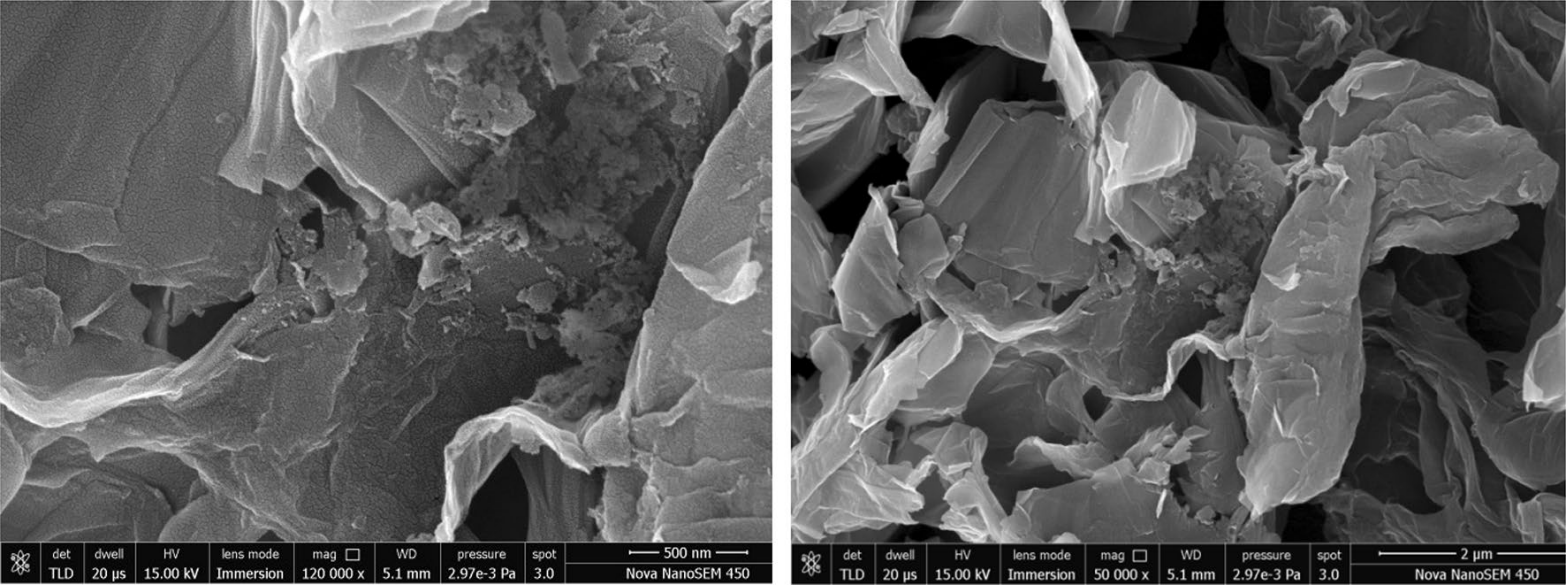
Field Emission Scanning Electron Microscopy (FESEM) of CuO–rGO.

FESEM was used to observe the catalyst’s dimensions and surface morphology. The pictures clearly show that the diameter of CuO is between 6 and 10 m, and deposition of CuO on the surface of rGO which corresponds to CuO nanoparticles, which are produced by rod-like shapes and some spherical shapes with a typical thickness of approx. 20 nm and a length of 75 nm. The CuO particles are decorated on the fringes of Graphene Oxide sheets. The existence of GO layers is indicated by the lamellar sheets. On rGO lamellar sheets, the rod-shaped CuO nanoparticles are attractively decorated and ornamented. Between CuO and rGO, a sufficient interfacial contact is formed. The charge transfer between CuO and rGO is encouraged by this type of interfacial interaction, as seen in the (Fig. 8) image.

**Figure 9:**
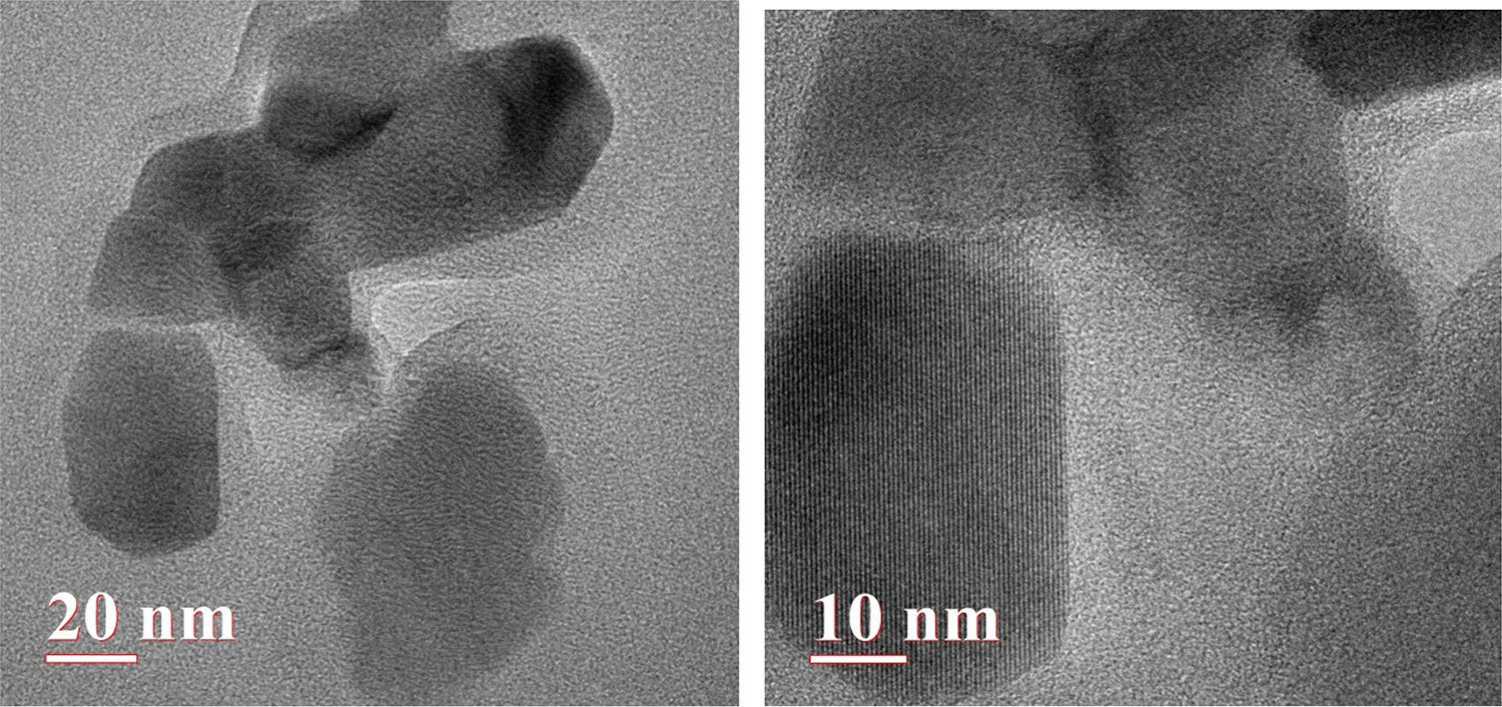
TEM images of rGO–CuO.

*TEM:* The TEM images of the synthesized sample are displayed below in Fig. 9. These images provided details on the size and shape of the composite. The blackish spots on the lamellar sheets of the Graphene oxide sheets are particles of Copper oxide. The images below also demonstrate the dispersion of particles and crystalline nature of particles. The spherical shape of Copper oxide is clealy visible in the picture below in the high magnification TEM image. The lattice fringes exhibit an interplanar lattice which corresponds to monoclinic lattice of CuO. The particle size was observed to be in the range of 18 nm to 45 nm.

**Figure 10:**
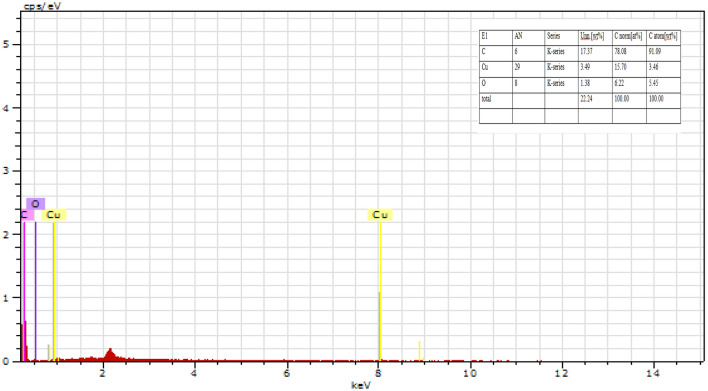
EDS of CuO–rGO.

*EDX/EDS Study of *CuO–rGO*:* The produced rGO–CuO nanocomposite's morphological characteristics and elemental composition are revealed by the Energy-Dispersive Spectroscopy (EDS) investigation shown in Fig. 10. The elemental composition of the rGO–CuO nanocomposite is revealed by the EDS spectrum, which also confirms the creation of a heterostructured hybrid structure made of CuO and rGO. Additionally, the elemental mapping supported the evenly arrangement of Copper Oxide on the support of GO in the picture below.

### Catalytic reduction reaction of nitroaromatics and dye degradation

Application of composite in the Catalytic Reduction Reactions of dye and remediation of nitro compounds. The nitroaromatic compounds have electron-withdrawing character which give them strong xenobiotic properties and in turn make them difficult to biodegrade. So, di and tri nitro compounds are difficult to biodegrade. The nitroaromatic compounds have been used in numerous chemical reactions and they have wide applications in drug, pesticides, insecticides, and corrosion industries. With noticeable applications they are threat to the mother earth also. As an industrial effluent, they can pollute water bodies and have carcinogenic effects on human beings also. In this section, some remediation techniques are discussed for the degradation of nitroaromatics such as 4-Nitroaniline, Picric acid and Nitrobenzene. Due to their distinctive electron-withdrawing character, nitro compounds have a highly deficient π-electron system, by generating a strong xenobiotic character. Nitroaromatic and other nitrogen containing organic compounds are resistant to biodegradation. Due to poor electron density on nitro group the electrophilic attack on aromatic ring becomes difficult which is the first step in the degradation^1^.

The catalytic performance of prepared material (CuO–rGO) was observed with the reduction of nitroaromatic compounds like Picric acid, 4-nitroaniline, Nitrobenzene and dyes. Relative nitro compounds was dissolved in hot water and prepared GO material was added in catalytic amount. After that, sodium borohydride was added in it and allowed to stir at RT till the completion of reaction. The monitoring of catalytic reduction reaction is checked by the TLC. We have also taken the UV visible graph of relative catalytic reaction to observe λ_max_.

*Reduction of 4-Nitroaniline (4-NA):* Aniline has large number of applications in the in industries such as textile industry, Drug industry, Corrosion and many more. However it is noticeable that the derivatives of aniline such as 4-Nitroaniline if released in the water bodies without proper treatment can have severe bad effects on mother nature and obviously human beings. Studies revealed that they can cause kidney and liver failure, eye irritation, also aniline derivatives have carcinogenic and mutagenic effects at low concentrations. So, proper treatment before releasing them in the water bodies is essential^41,42^.

**Figure 11:**
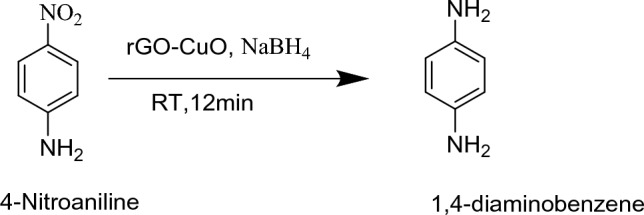
Reduction of 4-nitroaniline.

**Figure 12:**
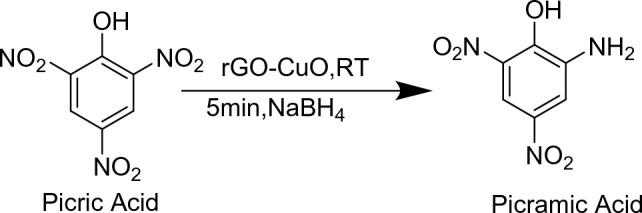
Reduction of picric acid.

**Figure 13:**
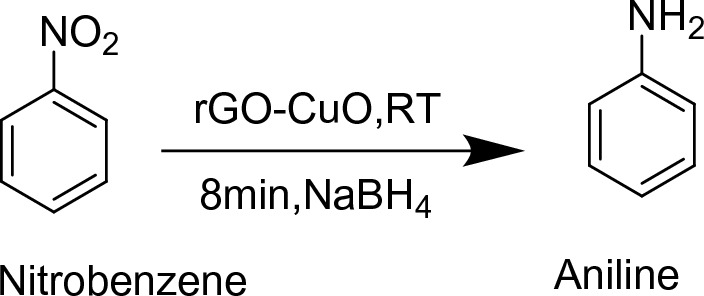
Reduction of nitrobenzene.

**Figure 14:**
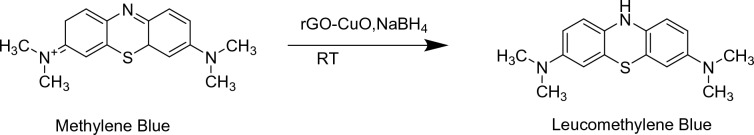
Reduction of Methylene Blue.

A comparative study of reduction of 4-nitroaniline was performed with three different types of prepared materials (CuO–rGO, Ag-rGO and NiO-rGO). We have also using different quantity of prepared material and Sodium Borohydride (9.5 mg, 19 mg, 28.5 mg) for the reduction of 4-Nitro aniline (Table 1). As shown in Table 1, the catalytic amount was varied from 5 to 15 mg of prepared material of each metal (CuO, Ag and NiO) deposited reduced graphene oxide for reduction of 4-Nitroaniline (Fig. 11). CuO–rGO (5 mg) is provided 100% reduction of 4-NA in 20 min, while 5 mg of each Ag-rGO and NiO-rGO provided in 28 and 90 min respectively (Table 1, entry 1 & 2). We have also varied the amount of CuO–rGO from 10 to 15 mg and observed that, relative catalytic product was found after 15 and 12 min respectively. The quantity of NaBH_4_ is also varied from 19 to 28.5 mg and catalytic reaction required 15 min and 12 min time respectively. We have also performed statistical analysis on the basis of this data and found that the reduced Copper oxide and Graphene oxide nanocomposite is better catalyst than the other two. So, we have used this nanocomposite in the catalytic reduction of other pollutants such as Picric acid (Fig. 12), Nitrobenzene (Fig. 13) and Methylene Blue dye (Fig. 14) also. (Table 1, entry 9–11)^43^.

**Table 1:** Optimization table for reduction of nitroaromatic compound.

Sr. No.	Catalyst	Catalyst loading (mg)	NaBH_4_ (mg)	Time (min)	% reduction (%)
1	rGO–NiO	5	9.5	38	100
2	rGO–Ag	5	9.5	90	100
3	rGO–CuO	5	9.5	24	100
4	rGO–CuO	10	9.5	18	100
5	rGO–CuO	15	9.5	10	100
6	rGO–CuO	5	19	20	100
7	rGO–CuO	10	19	13	100
8	rGO–CuO	15	19	9	100
9	rGO–CuO	5	28.5	16	100
10	rGO–CuO	10	28.5	10	100
11	rGO–CuO	15	28.5	6	100

From the optimization Tables 1 and 2 below it is clear that with increment in the amount of sodium borohydride and amount of catalyst loading the reduction time decreases drastically. Entry 5 shows that we have used increased the amount of catalyst loading i.e. 15 mg and kept sodium borohydride minimal i.e. 9.5 mg the reduction occurred in just 10 min. Similarly in Entry 9 we have kept amount of catalyst 5 mg and increased the amount of sodium borohydride i.e. 28.5 mg then also the reduction occurred in just 16 min. It clear that even 5 mg catalyst is sufficient for the reduction of nitroaromatic compound. The progress of reactions were observed using thin layer chromatography and it clearly shows that reduction has occurred with all the variations of catalyst loading and sodium borohydride.

**Table 2:** Comparison study of different catalyst with different parameters.

catalyst	cat. Loading (mg)	Water (ml)	4-Nitroaniline (mg)	Sodium borohydride (mg)	Time (min)
GO-NiO	5	10	14	9.5	38
GO-Ag	5 mg	10	14	9.5	90
GO-CuO	5	10	14	9.5	24
GO- NiO	10	10	14	9.5	23
GO-Ag	10	10	14	9.5	50
GO-CuO	10	10	14	9.5	18
GO-NiO	15	10	14	9.5	15
GO-Ag	15	10	14	9.5	15
GO-CuO	15	10	14	9.5	10
GO-NiO	5	10	14	19	25
GO-Ag	5	10	14	19	53
GO-CuO	5	10	14	19	20
GO-NiO	10	10	14	19	25
GO-Ag	10	10	14	19	38
GO-CuO	10	10	14	19	13
GO-NiO	15	10	14	19	15
GO-Ag	15	10	14	19	38
GO-CuO	15	10	14	19	9
GO-NiO	5	10	14	28.5	30
GO-Ag	5	10	14	28.5	15
GO-CuO	5	10	14	28.5	16
GO-NiO	10	10	14	28.5	20
GO-Ag	10	10	14	28.5	15
GO-CuO	10	10	14	28.5	10
GO-NiO	15	10	14	28.5	20
GO-Ag	15	10	14	28.5	15
GO-CuO	15	10	14	28.5	6

*Reduction of picric acid:* The nitroaromatic compounds have electron-withdrawing character which gives them strong xenobiotic properties and in turn make them difficult to biodegrade. So, di and tri nitro compounds are difficult to biodegrade. The yellow nitroaromatic compound Picric acid is a nitroaromatic compound having numerous applications in manufacturing of various industrial products such as dyes, pharmaceuticals and explosives etc^44,45^. They are classified as hazardous chemicals because of their bad effects on mother nature. As we know picric acid is used for making explosives and it is strong xenobiotic substance due to which it causes pollution in the industrial and military sites. The Trinitrophenol(TNP), is also difficult to degrade using microbes if the concentration of the pollutant is high due to the presence of three nitrocompounds. This nitroaromatic compound also have carcinogenic metabolites and can cause nausea, toxic hepatitis and death if the concentration is more than 5 mg/kg^45,46^. So, it is the need to reduce this nitro compound using safer and less hazardous means and below is the attempt to degrade 2,4,6-trinitriphenol using reduced Copper oxide and Graphene oxide composite as shown in Fig. 12. CuO–rGO (15 mg) was added in slightly heated aqueous solution of picric acid (0.1 mmol) and allowed to cool down till room temperature and stirred for 10 min. After that, solution of NaBH_4_ (28.5 mg) in 1 ml of distilled water was added in it and allowed to stirred at room temperature and progress of reaction was checked after every 2 min using thin layer chromatography^43^.

*Reduction of Nitrobenzene:* Nitrobenzene is one of the simplest nitro compound having large applications in industries in the manufacturing of aniline, and other organic chemicals. Its properties include light yellow colour, toxic liquid having bitter odour. Studies reveal that Nitrobenzene and derivatives can cause severe health effects methemoglobinemia which is a blood related disorder, anaemia, nausea, dizziness. In animals it can affect the reproductive organs, and in human beings International Agency for Research on Cancer reported that it can cause cancer^46,47^. Therefore it is classified as human carcinogen chemical by United States Environmental Protection Agency. Below is the reduction of Nitrobenzene into aniline(Fig. 13) using reduced Copper oxide and Graphene oxide composite and we have also discussed the bad environmental aspects of aniline above and it is also degraded using the same catalyst.

CuO–rGO (15 mg) was added in aqueous solution of nitrobenzene (0.1 mmol) and stirred for 10 min on magnetic stirrer. After that, the solution of NaBH_4_ (28.5 mg) in 1 ml distilled water was delivered into the reaction mixture and progress of reaction of reaction was checked after every 2 min using thin layer chromatography^48^.

*Reduction of dye(methylene blue):* Methylthioninium Chloride is also known as Methylene blue(thiazine dye). It has vast applications in textiles industry and drug industry. However its applications are considerable but its hazards are also seeking interest of researchers. As research specify the textile industries releases large amount of untreated dye in the water bodies which can cause serious health effects on aquatic life, human beings and animals^49^. It has toxic properties, can cause cancer, respiratory distress, can cause serious environmental issues and also it is non-biodegradable^50,51^.

CuO–rGO (10 mg) was added to the dye Methylene Blue, which was dissolved in water and stirred for 10 min, then 9.5 mg of NaBH_4_ was dissolved in 1 ml distilled water and added into it and again the reaction mixture was stirred. On addition of NaBH_4_ with continuous stirring, the colourful relative dye solution turns colourless after 2 min. UV–Visible spectroscopy confirms that the dye has degraded^51,52^. Below Fig. 14 shows the reduction of Methylene Blue dye into Leucometh-ylene Blue.

### UV Visible study of catalytic reduction reaction

**Figure 15:**
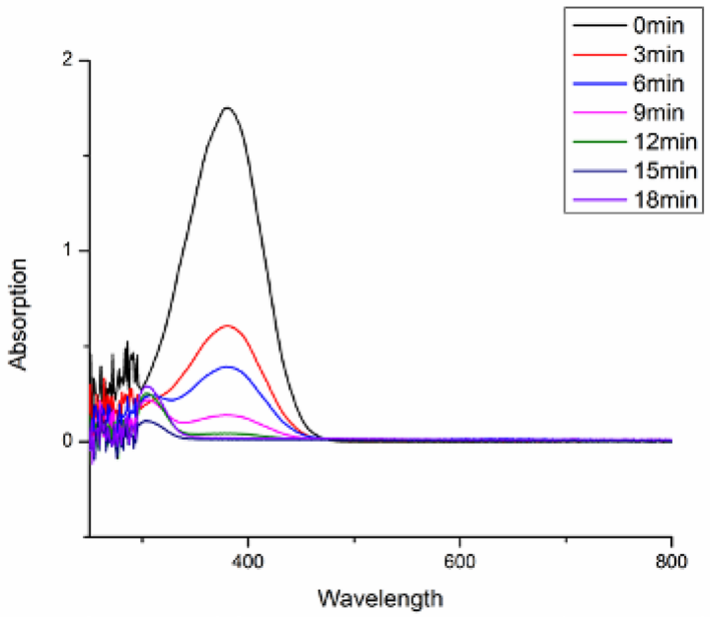
UV spectrum of 4-nitroaniline.

**Figure 16:**
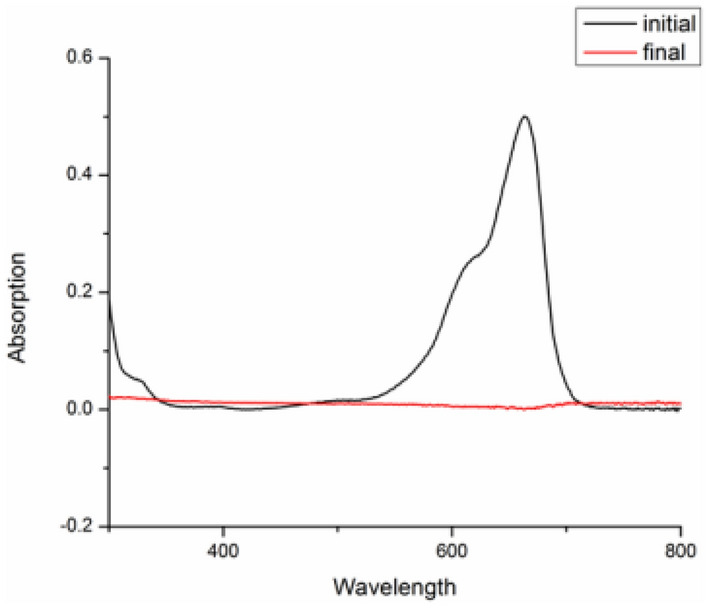
Degradation of methylene blue dye.

**Figure 17:**
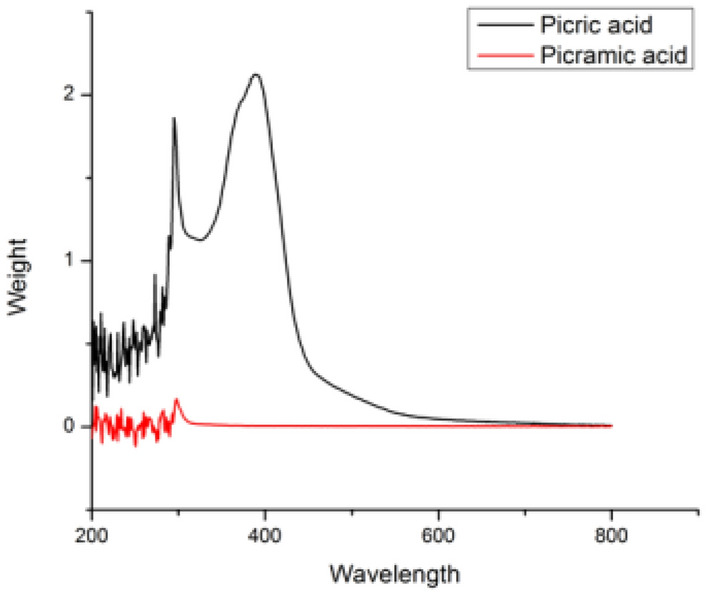
UV spectrum of reduction of Picric acid.

Reductive and degradation properties of rGO–CuO for dyes and nitroaromatic compounds were studied using the spectrophotometer (Figs. 15, 16, 17). Approx. 3 mL of sample (0.04 mM) for dye and nitroaromatics was taken in the cuvette and the UV–visible spectra were studied. The range taken was 200–800 nm. Initially a maxima was observed for 4-nitroaniline at 388 nm (Fig. 15). After complete reduction of 4-NA, maxima shift at 310 nm. Similarly UV spectra of Picric Acid has been taken (Fig. 17). For dye Methylene Blue (Fig. 16), a maxima was observed at 620 nm, which is disappeared after complete reduction.

*Statistical full factorial analysis* Full factorial experiment is done using analysis of variance technique by using SPSS version-26 in which reaction time (T) is taken as dependent variable and Sodium Borohydride (N), catalyst loading (CT), type of catalyst (C) are taken as independent variables at different levels. The benefit of statistical analysis is to compare the effect of various combinations of levels of sodium borohydride(factor-1) levels of catalyst loading(factor-2) and levels of type of catalyst(factor-3) on the reaction time. The analysis of variance techniques provides the individual effects of levels of each factor, interaction effects of two factors and interaction of three factors. By using software SPSS the results obtained are shown in Tables 3 and 4. For the analysis purpose levels of sodium borohydride, catalyst loading and type of catalyst are independent variables and time is dependent variable.

**Table 3:** The analysis of variance table in between subjects effects.

Source	Type III Sum of Squares	df	Mean Square	F	Sig
Dependent variable: T( time of reaction)					
Corrected model	21,058.469^a^	26	809.941	830.446	0.000
Intercept	56,379.864	1	56,379.864	57,807.203	0.000
N(NaBH_4_)	2919.432	2	1459.716	1496.671	0.000
CT(catalyst loading)	2978.840	2	1489.420	1527.127	0.000
C(type of catalyst)	4708.840	2	2354.420	2414.025	0.000
N * CT(NaBH_4_,* catalyst loading)	2688.864	4	672.216	689.234	0.000
N * C(NaBH_4_*type of catalyst)	3672.198	4	918.049	941.291	0.000
CT * C(catalyst loading*type of catalyst)	1486.346	4	371.586	380.994	0.000
N * CT * C(NaBH_4_*catalyst loading*type of catalyst)	2603.951	8	325.494	333.734	0.000
Error	52.667	54	0.975		
Total	77,491.000	81			
Corrected total	21,111.136	80			

^a^R Squared = .998 (Adjusted R Squared = .996)

**Table 4:** Post Hoc tests.

	(I)	(J)	Mean difference (I-J)	Std. error	Sig	95% confidence interval	
						Lower bound	Upper bound
Multiple comparisons							
Dependent variable: T (type of reaction)							
Tukey HSD for sodium borohydride	0.00	1.00	4.4815*	0.26878	0.000	3.8337	5.1292
		2.00	14.3704*	0.26878	0.000	13.7226	15.0181
	1.00	0.00	− 4.4815*	0.26878	0.000	− 5.1292	− 3.8337
		2.00	9.8889*	0.26878	0.000	9.2411	10.5367
	2.00	0.00	− 14.3704*	0.26878	0.000	− 15.0181	− 13.7226
		1.00	− 9.8889*	0.26878	0.000	− 10.5367	− 9.2411
Turkey HSD for catalyst loading	0.00	1.00	7.6667	0.26878	0.000	7.0189	8.3144
		2.00	14.8519	0.26878	0.000	14.2041	15.4996
	1.00	0.00	− 7.6667	0.26878	0.000	− 8.3144	− 7.0189
		2.00	7.1852	0.26878	0.000	6.5374	7.8330
	2.00	0.00	− 14.8519	0.26878	0.000	− 15.4996	− 14.2041
		1.00	− 7.1852	0.26878	0.000	− 7.8330	− 6.5374
Turkey HSD for type of catalyst	0.00	1.00 2.00	− 14.2963 3.2593	0.26878 0.26878	0.000 0.000	− 14.9441 2.6115	− 13.6485 3.9070
	1.00	0.00 2.00	14.2963 17.5556	0.26878 0.26878	0.000 0.000	13.6485 16.9441	14.9441 18.2033
	2.00	0.00 1.00	− 3.2593 − 17.5556	0.26878 0.26878	0.000 0.000	− 3.9070 − 18.2033	− 2.6115 − 16.9078

Individual effects of each parameter- for Sodium Borohydride if we consider three levels of NaBH_4_ i.e. 9.5 mg, 19 mg and 28.5 mg. Then, 28.5 mg gives the minimum reaction time. For catalyst loading if we consider three levels of them i.e. 5 mg, 10 mg, 15 mg. Then, 15 mg gives the minimum reaction time. For type of catalyst if we consider three levels i.e rGO–NiO, rGO–Ag, rGO–CuO. Then, rGO–CuO gives the minimum reaction time respectively.

Pairwise effects of parameters- for the pair of Sodium borohydride and catalyst loading, it was found that minimum reaction time occurred when we take catalyst loading as 15 mg and Sodium borohydride as 9.5 mg. For the pairwise interaction of Sodium borohydride and type of catalyst, minimum reaction time occurred when type of catalyst is rGO–CuO and amount of Sodium borohydride is taken as 28.5 mg. For the pairwise interaction of type of catalyst and catalyst loading, it was found that minimum reaction time occurred when we take type of catalyst as rGO–CuO and catalyst loading as 15 mg.

Three level interaction three parameters i.e. type of catalyst, amount of catalyst, amount of sodium borohydride gives minimum reaction time when type of catalyst is rGO–CuO, amount of catalyst is 5 mg and 15 mg and amount of sodium borohydride is 28.5 mg.

### Statistical analysis of catalytic reaction

Statistical studies of different catalysts and their derivatives like metal (CuO, Ag and NiO) deposited reduced Graphene oxide, Sodium borohydride, amount of catalyst was conducted at different levels and reaction time was also observed. Amount of Sodium Borohydride (NaBH_4_ ) were taken 9.5 mg, 19.5 mg, 28.5 mg. Amount of catalyst were taken 5 mg, 10 mg, 15 mg respectively.

There are three factors at three different levels. So, there will be 27 treatment combinations were analyzed. These 27 treatment combinations are repeated three times. So, total number of observations of different reaction time is obtained which is 81. The design of the experiment is full factorial experiment from which following objectives are obtained.

Objectives:

1. To test the individual effects of the parameters NaBH_4_(N), Amount of catalyst (CT), type of catalyst (C) on the reaction time (T).
2. To test the pairwise interaction between these three parameters type of catalyst, amount of catalyst and amount of sodium borohydride.
3. To test three factor interaction among the parameters type of catalyst, amount of catalyst and amount of sodium borohydride.
4. To identify the best combinations among the levels of parameters.

For achieving the above objectives following hypothesis are constructed-

i. To test the individual effects of the parameters NaBH_4_(N), Amount of catalyst(CT), time (T), type of catalyst(C).
  Null(H_0_): There is no significant different among various catalyst, amount of catalyst, NaBH_4_, with respect to reaction time.
  Alternative(H_1_): There is a difference among various catalyst, amount of catalyst, NaBH_4_, with respect to reaction time.
ii. To test the pairwise interaction between these three parameters type of catalyst, amount of catalyst and amount of sodium borohydride-
  H_0_: There is no pairwise significant difference between various catalyst, amount of catalyst, NaBH_4_, with respect to reaction time.
  H_1_: There is a difference among the pairs type of catalyst and Time, Sodium borohydride and amount of catalyst, type of catalyst and Sodium borohydride i.e.. various catalyst, amount of catalyst, NaBH_4_, with respect to reaction time.
iii. To test three factor interaction among the parameters type of catalyst, amount of catalyst, Sodium borohydride.
  H_○_: There is no significant different among the interaction of various catalyst, amount of catalyst, NaBH_4_, with respect to reaction time.
  H_1_: There is a difference among the three factor interaction among the parameters type of catalyst, amount of catalyst, Sodium borohydride.
iv. To identify the best combinations among the levels of parameters-
  The best combinations are shown when type of catalyst(C) is rGO–CuO, catalyst loading is 5 mg and 15 mg , amount of NaBH_4_ is 28.5 mg and respectively with respect to reaction time.

From the above Table 3 it is observed that there is a significant effect of type of catalyst, amount of catalyst, Sodium borohydride. Pairwise interaction among type of catalyst, amount of catalyst, Sodium borohydride because p values for all are 0.00 i.e. less than 0.05. Similarly three factor interactions type of catalyst, amount of catalyst and Sodium Borohydride is also significant.

Since all the factors, two factor interactions and three factor interactions show significant effect on time of reaction. So, after full factorial analysis of variance a post hoc test is conducted as shown in Table 4, and it was found that there is a significant difference among different levels of type of catalyst, amount of catalyst and Sodium Borohydride. It is concluded from the post hoc test that 28.5 mg of NaBH_4_ gives the minimum reaction time and also shows the significant difference with 9.5 mg and 19 mg. Similarly, 15 mg of catalyst loading gives the minimum reaction time and also shows the significant difference with 5 mg and 10 mg. If we consider, type of catalyst then, rGO–CuO gives the minimum reaction time and shows significant difference with rGO–NiO, rGO–Ag respectively. Similarly, Post hoc test is also applied on various levels of two factor interactions, it is found that two levels interaction of Sodium borohydride and Catalyst loading that is 9.5 mg and 15 mg shows the significant difference with other combinations of levels of two factor interactions. So, 9.5 mg of Sodium borohydride and 15 mg of Catalyst loading provides the minimum reaction time. Next, it is also found that two factor levels of Sodium borohydride and type of catalyst that is 9.5 mg of Sodium borohydride and rGO–CuO shows the significant difference with other combinations of levels of two factor interactions. So, 9.5 mg Sodium borohydride and rGO–CuO Catalyst provides the minimum reaction time. Next, it is found that two levels interaction of Catalyst loading and type of catalyst that is 15 mg catalyst loading and rGO–CuO shows the significant difference with other combinations of levels of two factor interactions. So, 15 mg catalyst loading and rGO–CuO catalyst provides the minimum reaction time respectively. Similarly if we consider three factor interactions at different levels the best combinations is types of catalyst rGO–CuO, catalyst loading is 5 mg or 15 mg and amount of NaBH_4_ is 28.5 mg respectively.

## Conclusion

A comparative study of the prepared composites of GO with different metal ion like Copper, Nickel and Silver has been done. Among these composites, Copper based graphene material (rGO–CuO) has been observed for best result provided and further it characterized by (FTIR), (PXRD), (TGA). (SEM) is used to study the surface morphology of these nanocomposites, and SEM images clearly shown the deposition of metals on Graphene Oxide, FTIR shows the presence of CuO, GO and also some amount of Cu_2_O in the composite of rGO–CuO, TGA confirms around 38% of the weight was lost dramatically after 150 °C, which may be related to the breakdown of the several functional groups that CuO–rGO contains and PXRD graph assured the crystalline structure of CuO–rGO was confirmed with powder, EDS shows the elemental composition are revealed by the energy-dispersive spectroscopy (EDS) investigation. The elemental composition of the rGO/CuO nanocomposite is revealed by the EDS spectrum, which also confirms the creation of a heterostructured hybrid structure made of CuO and rGO, TEM analysis confirm the preparation of nanomaterial and UV studies showed the reduction of nitroaromatics and degradation of Methylene Blue dye. Also it was found from the wet lab work methods and statistical analysis that three level interaction three parameters i.e. type of catalyst, amount of catalyst, amount of sodium borohydride gives minimum reaction time when type of catalyst is rGO–CuO, amount of catalyst is 5 mg and 15 mg and amount of Sodium borohydride is 28.5 mg which proves that rGO–CuO composites is showing better catalytic applications in the curtailment of 4-nitroaniline, Picric acid, Nitrobenzene and Methylene Blue dye respectively.

## Data Availability

The datasets used and/or analysed during the current study available from the corresponding author on reasonable request.
